# Validation study of a wellbeing scale (SPANE) in the Arab Gulf region: A multicountry study

**DOI:** 10.1371/journal.pone.0268027

**Published:** 2022-05-16

**Authors:** Saad Yaaqeib, Louise Lambert, Stavros Hadjisolomou, Manal Al-Fazari, Heyla Selim, Amber Haque

**Affiliations:** 1 Department of Cognitive Sciences, UAE University, Al Ain, UAE; 2 Department of Psychology, Canadian University Dubai, Dubai, UAE; 3 Department of Social and Behavioral Sciences, American University of Kuwait, Salmiya, Kuwait; 4 Department of Psychology, Sultan Qaboos University, Muscat, Oman; 5 Department of Psychology, King Saud University, Riyadh, Saudi Arabia; 6 School of Psychology and Social Work, Doha Institute for Graduate Studies, Doha, Qatar; Universita degli Studi di Firenze, ITALY

## Abstract

The Scale of Positive and Negative Experience (SPANE) is an emerging wellbeing scale to measure the frequency of positive and negative emotions. This study explores the psychometric properties of SPANE on a sample from the Arab Gulf region. The Arab Gulf region shares cultural elements with the broader Muslim and Arab world, but maintains distinct features that warrants validation studies for psychological instruments. There were 1393 participants from Saudi Arabia, Oman, Kuwait and other Arab Gulf countries. The factorial structure of SPANE was examined using a principal axis factor analysis, followed up with a confirmatory factor analysis. The convergent validity was examined by correlating SPANE with the Satisfaction with Life Scale (SWLS). The findings confirmed a two-factor structure of SPANE, and demonstrated adequate psychometric properties and convergent validity. In conclusion, this study indicates that SPANE shows sufficient validity for use as a measure of wellbeing in the Arab Gulf region.

## Introduction

Prior to the COVID-19 pandemic, wellbeing and mental health were an area of priority across the globe and this was no less the case in the Middle East/North Africa (MENA) region. The MENA region experiences a higher burden of mental health disorders than the global norm in adults and children alike [1–3], and this was also the case during the COVID-19 pandemic [4]. Going forward, this elevated burden will necessitate greater research efforts into providing psychological services that are both effective and culturally appropriate [5–7]. This further becomes an imperative in light of the fact that work over the years has revealed much psychological research suffers from bias; namely, it is Western in nature with treatments, measures, as well as understandings of wellbeing itself influenced by WEIRD (Western, Educated, Individualist, Rich and Democratic) narratives [8]. This has implications not only for how wellbeing initiatives are developed, but how the numbers supporting those actions are derived. In other words, do the wellbeing measures represent what they are supposed to measure? This question is central to this study, as we explore the validity of the Scale of Positive and Negative Emotions (SPANE) [9], a measure of positive and negative emotions across a subset of MENA nations: Saudi Arabia, Qatar and Kuwait. These nations represent the Gulf Cooperation Council (GCC) region, which possesses unique cultural features that distinguishes it from the broader Arab world. In this study we argue towards categorizing them as a distinct grouping rather than part of the whole MENA ensemble that obscures important regional differences.

## Literature review

### What is wellbeing?

Scientifically derived constructs such as flourishing, subjective wellbeing, life satisfaction, psychological wellbeing, engagement, positive emotion, etc., are used as proxies for wellbeing [9–16]. These terms reflect a common, yet overlapping distinction in the field, that of hedonic and eudemonic wellbeing [17]. Hedonic wellbeing entails a maximization of pleasure and minimization of pain [18]; that is, a focus on increasing the frequency of positive emotions, conducive to wellbeing on their own [19], and decreasing negative emotions, which includes symptoms of depression and anxiety as examples. Alternatively, personal growth, the use of skills and talents towards meaningful pursuits reflects a eudaimonic tradition [20]. Both approaches contribute to an overall state of wellbeing, with one being more immediate, while the other is experienced over time [21, 22].

#### Wellbeing in the region

A growing focus on wellbeing in the GCC nations has significantly raised its profile; many studies have been published exploring how it can be successfully increased, which necessarily includes how to measure gains. A series of studies in the UAE, Kuwait and Saudi Arabia have explored a variety of positive psychology interventions (PPIs) and their impact on the wellbeing of youth in schools [23] and university students [24–26], as well as the general population [27], many of which included the SPANE as a measure of interest. Reviews of the positive psychology intervention literature were also conducted [28–31], all showing the field to be slowly growing, but in need of higher-quality regional studies, as well as more attention to the cultural adaptation of interventions and measures alike. Of note, none of the studies mentioned addressed the validity of the SPANE in the samples for which it was being used.

Concerns over cultural adaptation and validation of scales are not new. Raised in mainstream psychology, such concerns have since become issues in positive psychology as well. Indeed, a recent paper highlighting the need for the Gallup World Poll to include more culturally diverse views in its surveying of global wellbeing is one such example [32], suggesting that the current state of science on this topic is neither complete, nor exhaustive and in fact, not fully representative of other parts of the world. Regional echoes of the need for greater attention to cultural and religious specificities in both positive psychology research and practise have also been identified [33–36].

#### Validation of wellbeing measures

Given the overwhelming array of wellbeing measures currently in use (160 counted by the Organization for Economic Co-operation and Development [37] alone and an estimated eight new tools designed every five years since the 1980s [38, 39], the scope for a lack of cultural specificity and validity is immense. While researchers may opt to develop scales for their own populations, there is nonetheless merit in using the same scales globally. Comparability of data between populations and nations is only possible with well used and more popular existing measures, but these also stem from rigorous standards of validity and reliability in other populations, as well as strong theoretical models to support them, unlike many homegrown measures [40–42]. Thus, validating wellbeing measures in various parts of the world serves a legitimate purpose, especially that evidence from the literature suggest that psychological constructs may manifest differently in this region [43].

There is growing interest in affective research, which tend to prioritize high arousal versus low types of positive affect. Many Eastern cultures value low arousal positive emotions (like calmness and contentment) to a greater degree [44]. However, the dominance of Western research in psychological sciences [45] suggest that nuances in emotional expression from other parts of the world may be underrepresented. Joshanloo [46, 47] has suggested this may be due, in part, to a fear of happiness shown in many Eastern and Muslim populations and confirmed in UAE studies [24, 25], as well as different views on happiness and its expression altogether.

A number of wellbeing measures have been validated in the MENA region. For instance, the Keyes et al. [48] Mental Health Continuum-Short Form (MHC-SF) and the Flourishing Scale [49] were both validated in Arabic on Egyptian samples [50, 51]. Likewise, the Subjective Happiness Scale [52] has been translated into Arabic and found to be valid, reliable, and culturally appropriate in a sample of Lebanese college students [53]. While these represent important validation studies, few have been conducted in the GCC region itself, a subset of the larger MENA area, home to smaller, culturally distinct and more recently established nations than those in the broader region.

#### The GCC nations: A distinct subset of the MENA region

The MENA region is not monolithic and can be split into three distinct groups [54]. First, the ‘resource-rich and labour-abundant countries are characterized by significant oil production and consumption and have large populations. These countries include Algeria, Iraq, or Syria. Second, the resource-poor group are countries who are small producers of oil and gas like Egypt, Jordan, Lebanon, Mauritania, Morocco, or Tunisia. Finally, the resource-rich and labour-importing countries are large producers of oil and gas and have a significant population of expatriate workers. These countries are mainly represented by the GCC states: Bahrain, Kuwait, Oman, Qatar, Saudi Arabia, and the United Arab Emirates. The GCC states are further characterized as having smaller population than the other subregions, disproportionately high incomes and a more qualified work force that relies extensively on expatriate and migrant labour, higher quality education systems and overall, more politically stable governance systems [55].

These latter states share traditional values, oil-based economies, linguistic roots, religious orientations, political governance systems, historical trajectories and sociocultural narratives [56, 57], which are distinct to a significant degree from the broader Arab and Muslim spheres [58–60]. The common sovereign elements between these states were officially acknowledged through the formation of the Gulf Cooperation Council (GCC) in 1981, to facilitate the collective progress and development of this region.

Historically, the mental health landscape has not received adequate attention in the region, as the social stigma associated with the field has persisted throughout the rapid modernization of the GCC states [61, 62]. However, in recent years there has been a growing concern about wellbeing and mental health issues from policymakers, with initiatives like the UAE’s appointment of a Happiness minister and nation-wide wellness programs [63] to formalize efforts in this domain, and passing laws to protect rights of mental health patients [64]. As a result of this trend, there has been increased interest in the use of psychological instruments to measure various facets of mental health and wellbeing. As the mental health domain steadily gains momentum, it is beneficial to develop a repertoire of psychological instruments that are culturally validated within the GCC region. Bearing in mind the distinctions of the GCC region from the broader MENA region, the aim is to provide decision-makers with culturally anchored data in formulating relevant policies, and enhance the local capacity in mental health assessments. This study is a contribution towards the inventory of GCC-validated measures of wellbeing.

## Method

### Participants

Participants were recruited via participating co-authors institutions. Each sought ethical approval to collect data in their respective universities and recruited participants from their local university student body across a wide range of programs and colleges. The overall study was approved by the first and second authors’ institutional ethics review board (UAE University, Research Ethics Review Board, Approval #ERS_2018_5763). All participants gave their informed consent to participate. Data were collected throughout the month February 2019 to the second week of March 2020 (prior to the start of the COVID-19 pandemic, with the exception of 18 respondents answering after this date).

### Instruments

#### Scale of Positive and Negative Experience (SPANE) [9, 49]

This 12-item self-report questionnaire includes two subscales: positive and negative. There are six items that measure positive feelings and six items that measure negative feelings. Respondents rate how often they have experienced the feelings listed (e.g., positive, negative, good, bad) in the past four weeks. None of the items are reverse-scored. Ratings are made on a 5-point scale from 1 = “very rarely or never” to 5 = “very often or always”. The positive and negative subscales are scored independently. The summed positive (SPANE-P) score and the negative (SPANE-N) score have the same range of 6 to 30. The balance (SPANE-B) score is obtained by subtracting the negative score from the positive score, yielding a score with a range of -24-24.

The SPANE generally performs well in terms of reliability and convergent validity with other measures of emotion, wellbeing, happiness, and life satisfaction [49]. It is well used globally and has been validated in a number of international studies [65–69]. The Arabic translated version of SPANE was obtained through the original authors’ web page where it is available for download and free for research use.

#### Satisfaction with Life Scale (SWLS)

This widely used 5-item measure [70] assesses respondent’s overall judgment of their satisfaction with life. Items (e.g., “I am satisfied with my life”, “If I could live my life over, I would change almost nothing”) are rated on a 7-point scale with final scores ranging from 5 to 35. The final score is the sum of the responses across the 5-items. The neutral point of the scale is 20, with higher scores indicating greater life satisfaction. It demonstrated high internal consistency, while test-retest reliability and convergent validity are also high [71]. The Arabic version of SWLS was obtained from the authors who validated the measure in the GCC region [72].

Participants were also asked a series of demographic and miscellaneous questions, which included: age, gender, marital status, number of children, hours of sleep, minutes exercising per week, smoking status, and length of time studying at the institution.

#### Procedure

Participants were sent an email requesting their participation. In it, they were provided with a link to two written consent forms, one in Arabic and another in English (in compliance with the local Ethics Review Board guidelines) based on their language preference. Both forms contained identical texts, and both also included a contact name in English and Arabic should there have been any further questions. The consent page informed participants about their right to not take part in the study or complete it. Providing consent (by clicking an agreement tick-box) was necessary to be able to proceed. The survey (including SPANE and SWLS) was in the Arabic language. No class credit was given for participation. The statistical analysis was based on the salient groups that emerged in the sample. The two countries with largest number of respondents were Oman and KSA. Respondents from other GCC countries were consolidated into one group to avoid statistical power issues with small samples in confirmatory factor analysis (CFA), as recommended by Kyriazos [73].

#### Data analysis

The data analysis process included the following steps. First, the factor structure of SPANE was explored through a principal factor analysis (PFA). Second, a confirmatory factor analysis (CFA) was applied to assess the fit of the data to SPANE’s original factorial structure (including model fit statistics). Finally, convergent validity was established by investigating the correlation coefficients between SPANE scores and SWLS scores.

## Results

### Descriptive results and internal consistency

Table 1 presents the demographic characteristics of the study’s sample, as well as the wellbeing scale scores relevant to each group. Table 2 presents the mean values, standard deviations, and Cronbach alpha coefficients of the SWLS, SPANE-Positive, SPANE-Negative, and SPANE-Balance subscales. The Cronbach alpha coefficients ranged from 0.79 to 0.87, indicating high consistency.

**Table 1:** Descriptive statistics of the sample (N = 1393).

		SWLS			SPANE P		SPANE N		SPANE B	
		Count	Mean	SD	Mean	SD	Mean	SD	Mean	SD
**What is your age?**	Total	1393	23.73	6.81	17.68	3.27	16.65	4.17	4.52	7.23
	18–24	1183	23.61	6.82	17.64	3.29	16.78	4.17	4.32	7.28
	25–34	157	24.04	7.00	17.95	3.18	15.98	4.26	5.60	7.32
	35 or more	53	25.43	5.74	17.85	3.02	15.72	3.77	5.79	5.37
**What is your gender?**	Female	947	23.82	6.85	17.68	3.26	16.97	4.25	4.19	7.34
	Male	446	23.54	6.72	17.67	3.29	15.99	3.94	5.23	6.94
**Current relationship status?**	Married	139	25.37	5.87	18.23	3.24	15.72	4.15	6.17	7.29
	Widow	6	19.00	6.20	15.83	2.56	18.00	4.10	1.17	6.94
	Divorced	20	23.55	5.40	18.05	3.35	15.50	5.15	6.20	8.64
	In a relationship	100	23.12	7.45	17.70	3.09	16.70	3.91	4.54	6.93
	Single	1128	23.61	6.85	17.61	3.28	16.78	4.17	4.30	7.20
**Do you have children under 18?**	Yes	118	24.80	6.22	18.14	3.27	15.99	4.11	5.76	7.37
	No	1275	23.63	6.85	17.64	3.26	16.71	4.18	4.41	7.21
**How many hours of exercise every week?**	None	304	21.91	7.32	16.77	3.50	17.76	4.65	2.25	7.92
	30 Minutes	338	23.96	6.80	17.61	3.21	16.84	3.92	4.23	6.92
	30–60 Minutes	234	23.96	6.66	17.99	3.10	16.65	4.08	4.94	7.08
	60–90 Minutes	163	24.29	6.06	17.85	3.20	16.26	3.94	5.18	6.73
	90–120 Minutes	104	24.85	6.80	17.91	3.25	15.72	3.58	5.74	6.83
	120 or More	250	24.58	6.42	18.38	3.02	15.70	4.05	6.36	6.68
**How many hours of sleep do you get everyday?**	Less than 5	189	20.63	7.30	16.20	3.66	17.89	4.79	1.56	8.15
	Between 5 and 7	837	24.22	6.50	17.95	3.11	16.42	4.02	5.08	6.96
	Between 7 and 9	293	24.47	6.35	18.01	3.00	16.10	3.86	5.45	6.54
	More than 9	74	23.09	8.37	17.09	3.88	18.32	4.46	2.11	8.07
**Are you a smoker of any kind (tobacco, sheesha, etc.)?**	Yes, regularly	53	19.87	7.35	16.11	3.48	18.68	4.84	.64	8.23
	Yes, but only on occasion	62	19.60	8.14	16.23	4.02	18.50	4.49	.90	8.84
	No	1278	24.09	6.60	17.82	3.18	16.48	4.09	4.86	7.01
**Country**	Other GCC	119	24.22	6.79	17.95	3.32	17.15	4.59	4.35	7.58
	KSA	368	22.32	7.51	17.02	3.72	17.36	4.54	2.96	8.05
	Oman	906	24.24	6.43	17.92	3.02	16.30	3.92	5.18	6.73

**Table 2:** Summary scale statistics.

	n	Mean	SD	α
**SWLS**	1393	4.118	6.806	.860
**SPANE P**	1393	21.173	3.855	.834
**SPANE N**	1393	16.652	4.174	.790
**SPANE B**	1392	4.521	7.231	.871

### Factorial validity

The adequacy of the data for factor analyses was explored using the Kaiser-Meyer-Olkin (KMO) measure of sampling adequacy and the Bartlett’s test of sphericity. The KMO result was .917, indicating a high level of sampling adequacy. The Bartlett test result was significant (p < .001), indicating that a factor analysis is appropriate for the data structure.

SPANE’s factor structure was first examined using a principal factor analysis (PFA). Table 3 indicates that two factors emerged with an eigenvalue greater than 1.0, and these two factors accounted for approximately 53% of the total variance. This result supports the original bidimensional structure of the SPANE scale as developed by Diener et al. (2010). The factor loadings of the twelve items ranged from 0.46 to 0.76 (shown in Table 4).

**Table 3:** Principal axis factoring—Total variance explained.

Factor	Initial Eigenvalues		
	Total	% of Variance	Cumulative %
**1**	5.216	43.466	43.466
**2**	1.197	9.972	53.438
**3**	.790	6.580	60.018
**4**	.770	6.414	66.431
**5**	.713	5.940	72.372
**6**	.694	5.780	78.151
**7**	.640	5.335	83.486
**8**	.485	4.042	87.528
**9**	.448	3.732	91.260
**10**	.390	3.248	94.509
**11**	.351	2.928	97.436
**12**	.308	2.564	100.000

**Table 4:** Principal axis factoring—Factor loadings.

	Factor	
	1	2
**Joyful**	.761	-.268
**Happy**	.745	-.328
**Pleasant**	.711	-.253
**Positive**	.567	-.403
**Contented/Satisfied**	.464	-.223
**Good**	.446	-.220
**Sad**	-.344	.643
**Negative**	-.318	.638
**Bad**	-.322	.631
**Unpleasant**	-.329	.586
**Afraid**	-.187	.473
**Angry**	-.127	.459

**Fig 1:**
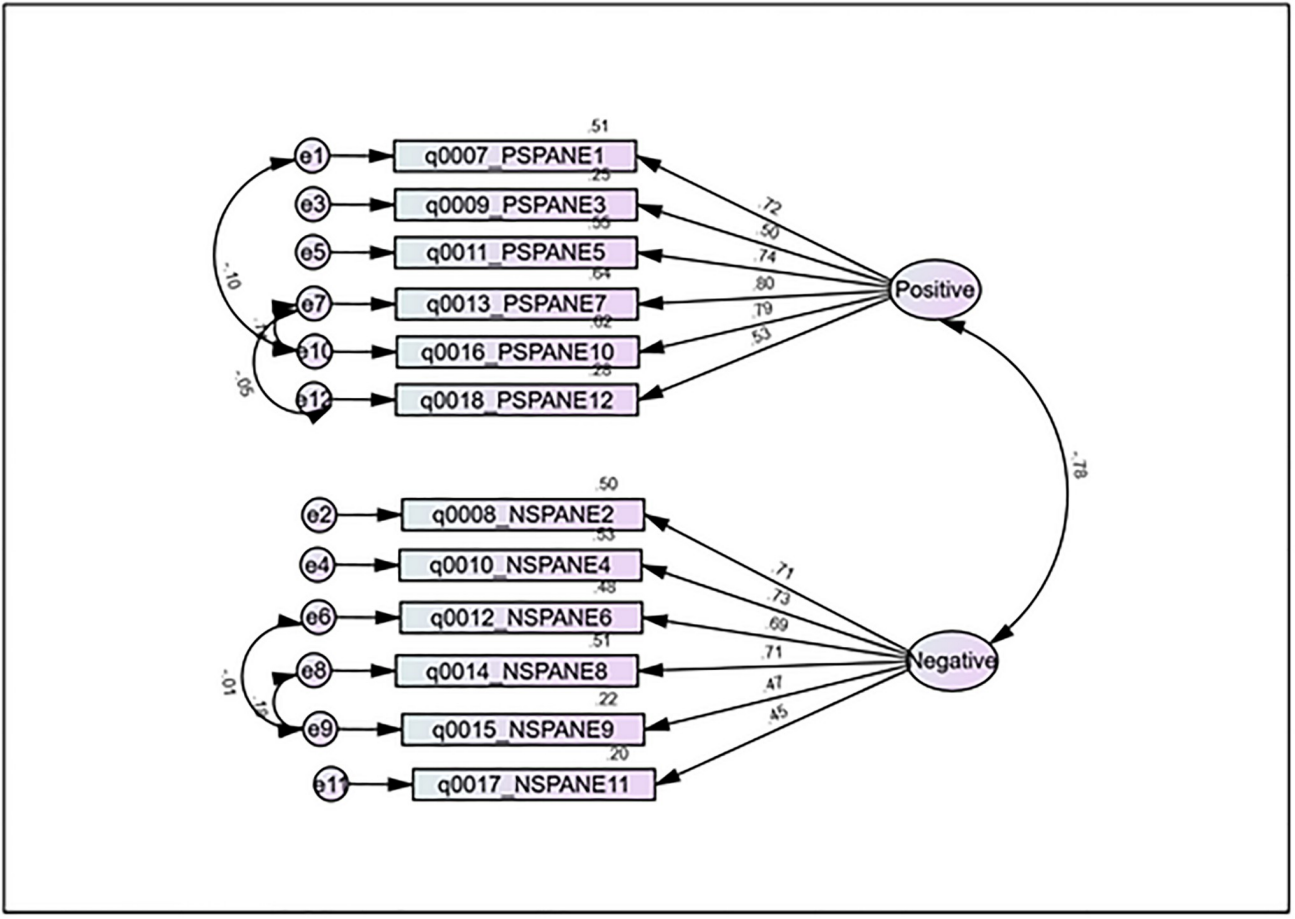
Graphical representation the two-factor model of SPANE as demonstrated by the confirmatory factor analysis. Loadings shown are standardized loading.

A confirmatory factor analysis (CFA) was conducted to test the fit of the data to the 2-factor structure. Table 5 indicates that all items loaded on the Positive feelings (Positive, Good, Pleasant, Happy, Content/Satisfied, Joyful) and Negative feelings (Negative, Unpleasant, Bad, Sad, Afraid, Angry) constructs. The standardized factor loadings ranged from 0.45 to 0.80. All the variables significantly loaded (p <0.01) into their respective constructs, indicating that the model possesses content validity. To improve the model fit, high covariances between items of the same factor were identified through SPSS Amos’s modification indices (indicated by a double-headed arrow between the errors in Fig 1).

**Table 5:** Confirmatory factor analysis—Factor loadings.

*Items*	Factor1 (Positive)	Factor2 (Negative)
**q0007_PSPANE1 (Positive)**	.716	
**q0009_PSPANE3 (Good)**	.498	
**q0011_PSPANE5 (Pleasant)**	.744	
**q0018_PSPANE12 (Content / Satisfied)**	.526	
**q0016_PSPANE10 (Joyful)**	.785	
**q0013_PSPANE7 (Happy)**	.798	
**q0008_NSPANE2 (Negative)**		.706
**q0010_NSPANE4 (Bad)**		.725
**q0012_NSPANE6 (Unpleasant)**		.691
**q0014_NSPANE8 (Sad)**		.712
**q0015_NSPANE9 (Afraid)**		.468
**q0017_NSPANE11 (Angry)**		.449

Table 6 presents the goodness of fit indices of the CFAs conducted. The *χ*^2^ statistic was significant across all the models tested, which generally indicates an inadequate model fit to the data. However, the *χ*^2^ statistic is sensitive to sample size [74], therefore it is usually taken into consideration with other model fit indices. The full sample CFA indicates that the two-factor model generally fitted the data, with the CFI, RMSEA and NFI indices being within desirable ranges. To test for measurement invariance across countries, a multi-group analysis was conducted where the two-factor model fit was simultaneously examined across the subgroups of Saudi Arabia, Oman and Other GCC. Table 6 shows that the full configural model had a good fit to the data, with all the goodness of fit indices to be within desirable or acceptable ranges (with the except of the RMSEA index which was slightly lower).

**Table 6:** Goodness of fit statistics of SPANE (CFA).

	n	χ2	df	χ2/df	CFI	SRMR	RMSEA	NFI
Desired Range*				**>3**	**>0.95**	**>0.08**	**>0.06**	**>.95**
**Full Sample**	**1393**	**276.325**	**48**	**5.757**	**.963**	**0.032**	**.058**	**.956**
**Oman**	**906**	**229.735**	**48**	**4.786**	**.953**	**.037**	**.065**	**.941**
**KSA**	**368**	**119.628**	**48**	**2.492**	**.960**	**.041**	**.064**	**.935**
**Other GCC**	**119**	**75.682**	**48**	**1.577**	**.952**	**.071**	**.070**	**.881**
**Multigroup**		**425.349**	**144**	**2.954**	**.955**	**.071**	**.037**	**.934**

These results suggest that the assumption of configural invariance was confirmed, and that it is safe to assume that the two-factor model of SPANE was supported across the three countries. However, as an extra precautionary measure, the CFAs were conducted on each country separately to evaluate the two-factor model fit (also shown in Table 6), demonstrating slightly varying levels of goodness of fit but overall supporting the two-factor structure of the Arab Gulf version of SPANE.

### Convergent validity

To explore convergent validity, the correlations between SPANE and SWLS were examined. Table 7 shows substantial correlations between the scales, with all of them being significant at p < 0.05. SWLS was negatively correlated with SPANE-Negative scores (r = -0.53). SWLS scores highly correlated with SPANE-Positive and SPANE-Balance scores (r = 0.648 and 0.653). Furthermore, the intercorrelations were also examined revealing expected patterns. SPANE-Negative demonstrated a high negative correlation with SPANE-Balance (r = -0.908) and was also negatively correlated with SPANE-Positive scores (r = -0.593).

**Table 7:** Correlations.

	**SPANE_P**	**SPANE_N**	**SPANE_B**	**SWLS**
**SPANE_P**	1	-.593**	.867**	.648**
**SPANE_N**	-.593**	1	-.908**	-.530**
**SPANE_B**	.867**	-.908**	1	.653**
**SWLS**	.648**	-.530**	.653**	1

**. Correlation is significant at the 0.01 level (2-tailed).

## Discussion

The purpose of this study is to explore the validity of the SPANE scale in the Arab Gulf region. Data obtained from GCC countries supported the two-factor structure of the original SPANE developed by Diener et al. [49]. The results demonstrated appropriate reliability, content validity, factorial validity, and convergent validity.

Furthermore, the multi-group CFA indicated the general structure was consistent across the three countries included in the sample, demonstrating strong measurement invariance [75]. In other words, the sample’s subgroups (countries) did not harbor systematic differences in the responses to the SPANE. This result is consistent with the similar sociocultural context shared by populations of GCC states.

This study contributes to literature of cross-cultural validation studies of the SPANE. The psychometric properties of the SPANE found in this study resonate with several cross-cultural validation studies from Portugal [68], Japan [69], China [65], Germany [67], and Spain [66]. It is a point of interest that samples from both individualist and collective cultures demonstrate similar willingness to identify positive and negative emotions when prompted, considering the differences in expressing emotions between these cultural architypes as documented by the literature [76–78].

In conclusion, the findings indicated that the SPANE exhibited similar psychometric properties to its original version [49] when applied in the Arab Gulf region using the Arabic language. The growing evidence of SPANE’s cross-cultural validity has significant implications for its universality.

Therefore, SPANE is a reliable and valid psychological instrument that can be employed by policy-makers, academics and practitioners in the GCC states for the development of wellbeing initiatives as well as mental health infrastructure and cultivation of mental health awareness. The versatility and ease of use makes it an appropriate scale to apply on a wide scale.

Overall, the results demonstrated appropriate reliability, content validity, factorial validity, and convergent validity. The nuances between countries suggest that there are within-group differences that may be worth exploring further.

### Limitations and future directions

The current study was conducted on a sample of students, which may not offer an accurate representation of the populations of the Arab Gulf countries. Therefore, there is a concern of the generalizability of the results, as the SPANE factorial structure may differ when applied on the general public. Another issue with the sample is the disproportionate distribution of participants across the sample subgroups. However, considering that the main survey was conducted online, there was limited control over the locations of the respondents.

As a follow up study, it may be worthwhile to investigate lower loading items like Happy/Content or Angry/Angry through a qualitative approach. The authors speculate that there may be social factors that hinder the population from identifying with particular emotions. Furthermore, the differences in SPANE levels between the subgroups (shown at the bottom of Table 1) in the study may offer venues for future research. In this study SPANE’s convergent validity was explored using the SWLS. Investigating SPANE along other measures of wellbeing may contribute towards its convergent and divergent validity.

## Supporting information

S1 File Survey data. The SPSS data of SPANE and SWLS in the Arab Gulf (applied on university students).
(SAV)
